# Flagellin Induces β-Defensin 2 in Human Colonic *Ex vivo* Infection with Enterohemorrhagic *Escherichia coli*

**DOI:** 10.3389/fcimb.2016.00068

**Published:** 2016-06-21

**Authors:** Steven B. Lewis, Alison Prior, Samuel J. Ellis, Vivienne Cook, Simon S. M. Chan, William Gelson, Stephanie Schüller

**Affiliations:** ^1^Norwich Medical School, University of East AngliaNorwich, UK; ^2^Gut Health and Food Safety Programme, Institute of Food ResearchNorwich, UK; ^3^Gastroenterology Department, Norfolk and Norwich University HospitalNorwich, UK

**Keywords:** EHEC, flagellin, colon, β-defensin, interleukin-8, inflammation

## Abstract

Enterohemorrhagic *E*.coli (EHEC) is an important foodborne pathogen in the developed world and can cause life-threatening disease particularly in children. EHEC persists in the human gut by adhering intimately to colonic epithelium and forming characteristic attaching/effacing lesions. In this study, we investigated the innate immune response to EHEC infection with particular focus on antimicrobial peptide and protein expression by colonic epithelium. Using a novel human colonic biopsy model and polarized T84 colon carcinoma cells, we found that EHEC infection induced expression of human β-defensin 2 (hBD2), whereas hBD1, hBD3, LL-37, and lysozyme remained unchanged. Infection with specific EHEC deletion mutants demonstrated that this was dependent on flagellin, and apical exposure to purified flagellin was sufficient to stimulate hBD2 and also interleukin (IL)-8 expression *ex vivo* and *in vitro*. Flagellin-mediated hBD2 induction was significantly reduced by inhibitors of NF-κB, MAP kinase p38 and JNK but not ERK1/2. Interestingly, IL-8 secretion by polarized T84 cells was vectorial depending on the side of stimulation, and apical exposure to EHEC or flagellin resulted in apical IL-8 release. Our results demonstrate that EHEC only induces a modest immune response in human colonic epithelium characterized by flagellin-dependent induction of hBD2 and low levels of IL-8.

## Introduction

Enterohemorrhagic *E. coli* (EHEC) is a foodborne pathogen of worldwide importance (Croxen et al., 2013). Although infections with EHEC are rare compared with *Campylobacter* and *Salmonella*, they can lead to severe systemic hemolytic uremic syndrome (HUS) resulting in kidney failure and death (Tarr et al., 2005). EHEC mainly affects young children and the elderly, and the predominant serotype in most parts of the world is O157:H7 (Croxen and Finlay, 2010).

EHEC causes diarrhea by adhering to human intestinal epithelium, particularly the colon, and forming attaching and effacing (A/E) lesions (Golan et al., 2011; Lewis et al., 2015). These are characterized by intimate bacterial attachment to the host cell membrane and effacement of underlying microvilli. A/E lesion formation is dependent on a bacterial type III secretion system (T3SS) which enables EHEC to inject effector proteins into the host cell (Jarvis and Kaper, 1996). Around 50 EHEC effector proteins have been identified so far (Tobe et al., 2006), affecting many different host cell functions such as maintenance of epithelial barrier integrity, water and ion transport, and immune response to infection (Viswanathan et al., 2009). In addition to the T3SS, EHEC also produces Shiga toxins (Stxs) which are linked to HUS and strongly cytotoxic to kidney cells (Obrig and Karpman, 2012). After release into the gut lumen, Stxs transverse the intestinal epithelium and are transported to the kidneys via the bloodstream (Schüller, 2011).

EHEC infection in the human gut is accompanied by neutrophil recruitment to the intestinal mucosa (Griffin et al., 1990; Kelly et al., 1990), and high levels of the neutrophil chemoattractant interleukin (IL)-8 have been detected in HUS patients (Fitzpatrick et al., 1992; Murata et al., 1998) indicating a key role of this cytokine in the innate immune response to EHEC infection. In addition to cytokine production which recruits phagocytes to the site of infection, secretion of antimicrobial peptides and proteins (AMPs) such as defensins, cathelicidins, and lysozyme by intestinal epithelium constitutes an important part in the innate immune defense against intestinal pathogens. Pore-forming AMPs directly kill bacteria and also promote an inflammatory immune response by acting as chemoattractants (Wassing et al., 2014). Human colonic epithelium, the primary target site of EHEC, produces lysozyme and LL-37, which is the only cathelicidin in humans and expressed by upper crypt epithelial cells. In addition, four types of defensins are secreted by human colonic epithelium: While human β-defensin (hBD)1 is constitutively expressed, hBD2-4 are induced by infection and inflammation (Eckmann and Kagnoff, 2005; Muniz et al., 2012).

Previous studies have demonstrated that infection with intestinal pathogens can modulate expression of AMPs and cytokines by the host epithelium, thereby resulting in an induced or suppressed immune response (Islam et al., 2001; Zilbauer et al., 2005; Sharma et al., 2006; Sperandio et al., 2008). In this study, we have investigated the influence of EHEC infection on AMP and IL-8 expression in physiologically relevant colonic biopsy and polarized cell culture models.

## Materials and methods

### Bacterial strains and flagellin

Bacterial strains used in this study are listed in Table 1. Bacteria were grown standing in LB broth overnight at 37°C. Deletion mutants were selected with kanamycin (50 μg/ml) or chloramphenicol (30 μg/ml). Bacteria were spun down before infection and suspended in serum-free culture medium. LPS-free purified H7 monomeric flagellin was kindly provided by David Gally, Roslin Institute, Edinburgh.

**Table 1:** ***E. coli* strains used in this study**.

**Name**	**Description**	**Source or references**
EDL933	Wild-type EHEC O157:H7	Riley et al., 1983
EDL933 Δ*escN*	EDL933 *escN* deletion mutant	Jarvis and Kaper, 1996
EDL933 Δ*fliC*	EDL933 *fliC* deletion mutant	Gobert et al., 2008
EDL933 Δ*stx*	EDL933 *stx1 stx2* deletion mutant	Gobert et al., 2007
85–170	Stx-negative EHEC O157:H7	Tzipori et al., 1987
85–170 Δ*hcpA*	85–170 *hcpA* deletion mutant	Xicohtencatl-Cortes et al., 2007
85–170 Δ*lpfA1*	85–170 *lpfA1* deletion mutant	Fitzhenry et al., 2006
Walla-1	EHEC O157:H7	Ostroff et al., 1990
H0-7184-0336	EHEC O157:H7	G. Smith, Public Health England

### Cell culture and infection

Human colon carcinoma T84 cells (ATCC CCL-248) were cultured in DMEM/F-12 mixture supplemented with 10% fetal bovine serum (Sigma) and used between passage 45 and 65. To obtain polarized monolayers, cells were seeded out on collagen-coated Transwell filter inserts (12 mm diameter, 0.4 μm pore; Corning Costar) at a density of 5 × 10^5^ cells/insert. Transepithelial electrical resistance (TER) was monitored using an EVOM2 resistance meter with an STX2 electrode (World Precision Instruments), and values above 1500 Ω × cm^2^ after 7–10 d of differentiation indicated establishment of epithelial barrier function. Polarized T84 cells were infected with approximately 6 × 10^7^ bacteria in plain DMEM/F-12 medium and incubated for 3 h. After that, medium was exchanged every hour to prevent bacterial overgrowth and acidification of the medium. For 24 h infections, gentamicin (200 μg/ml, Sigma) was added 1 h after infection to slow down bacterial growth, and incubations were continued without medium exchange for up to 24 h. For incubations with flagellin and IL-1β (Sigma), no medium exchange was performed. For signal transduction studies, cells were incubated with the chemical inhibitors quinazoline (NF-κB) (28 μM, Sigma), SB203580 (p38), SP600125 (JNK), or PD98059 (ERK 1/2) (25 μM, Merck Millipore) for 1 h before flagellin (0.1 μg/ml) was added for 6 h. Cells were incubated at 37°C in a 5% CO_2_ atmosphere. At the end of the experiment, cells were washed twice in PBS to remove non-adherent bacteria and processed according to further analysis.

### Polarized *In vitro* organ culture (pIVOC)

This study was performed with approval from the University of East Anglia Faculty of Medicine and Health Ethics Committee (ref 2010/11-030). All samples were provided through the Norwich Biorepository, which has NRES approval (ref 08/h0304/85+5). Up to four biopsy samples from the transverse colon were obtained with informed consent during colonoscopy of 23 adult patients (24–77 years old) during routine investigation of potential intestinal disorders. Samples were taken from macroscopically normal areas, transported to the laboratory in IVOC medium and processed within the next hour. Polarized IVOC was performed as described previously (Schüller et al., 2009). Briefly, biopsies were mounted mucosal side upwards on a circular cellulose nitrate filter and sandwiched between two Perspex disks with a 2 mm central aperture (manufactured by the School of Environmental Sciences workshop, University of East Anglia). To prevent bacterial leakage, the apical disk was sealed to the mucosal side of the biopsy with Histoacryl tissue glue (Braun Medical). The sandwich holding the biopsy was then mounted in a Snapwell support (Corning Costar) and inserted in a six-well culture plate. Apical and basal compartments were filled with IVOC medium, and 20 μl of bacterial overnight culture (~10^7^ bacteria), purified monomeric H7 flagellin or IL-1β were added apically. A specimen inoculated with medium only was included with each experiment to exclude *in vivo* bacterial colonization. Samples were incubated in a 5% CO_2_ atmosphere at 37°C on a rocking platform for 7 h. Bacterial leakage into the basolateral compartment was assessed by bacterial growth and turbidity of the medium, and respective samples were excluded from analysis. At the end of the experiment, biopsies were removed from the Snapwell support, washed in PBS to remove mucus and non-adherent bacteria and processed for further analysis.

### RNA isolation and quantitative real-time PCR (qPCR) analysis

Total RNA from cells and biopsies was isolated using the RNeasy Mini kit with on-column DNase digestion (Qiagen) according to the manufacturer's instructions. Biopsy tissue was homogenized with a pestle (Kimble Chase) before extraction. RNA quality was assessed by gel electrophoresis and OD_260∕280_ determination. RNA was quantified using a Nanodrop ND-1000 spectrophotometer, and 1 μg RNA was converted to cDNA using the qScript cDNA supermix (Quanta Biosciences) in a 20 μl reaction. Quantitative real-time PCR was performed using an ABI 7500 PCR system (Applied Biosystems). Primers were purchased from Sigma-Genosys. Gene-specific sequences (Table 2) were obtained from published studies or designed using PrimerBLAST software (http://www.ncbi.nlm.nih.gov/tools/primer-blast/). Up to 4 μl cDNA were amplified in a 10 μl reaction containing 0.5 μM of each primer and 5 μl of 2 × SYBR Green JumpStart Taq ReadyMix (Sigma). Cycling parameters were as follows: 2 min at 95°C (initial denaturation); 30 s at 95°C, 30 s at 60°C, 35 s at 72°C (40 cycles); 5 min at 72°C (final elongation); 15 s at 95°C, 60 s at 60°C, 15 s at 95°C, 15 s at 60°C (dissociation for melt curve analysis). PCR product specificity was confirmed by melt curve analysis and agarose gel electrophoresis. Relative quantification of gene expression was performed using the comparative Ct method. Ct values for genes of interest were normalized using the geometric mean Ct of two housekeeper genes, glyceraldehyde-3-phosphate dehydrogenase (GAPDH) and RNA polymerase II polypeptide A (POLR2A). Fold expression levels in treated samples were calculated relative to matched non-treated controls using the formula 2^−ΔΔCt^.

**Table 2:** **Primer sequences used in this study**.

**Target**	**Primer sequence**	**References**
hBD1	F 5′-CTGCTGTTTACTCTCTGCTTACTTTT-3′	Fahlgren et al., 2003
	R 5′-CCTCCACTGCTGACGCA-3′	
hBD2	F 5′-CTCGTTCCTCTTCATATTCCTGA-3′	Fahlgren et al., 2003
	R 5′-CTAGGGCAAAAGACTGGATGAC-3′	
hBD3	F 5′-TGAAGCCTAGCAGCTATGAGGATC-3′	Fahlgren et al., 2004
	R 5′-CCGCCTCTGACTCTGCAATAA-3′	
hBD4	F 5′-CCCAGCATTATGCAGAGACTT-3′	Fahlgren et al., 2004
	R 5′-ACCACATATTCTGTCCAATTCAAAT-3′	
IL-8	F 5′-TTGAGAGTGGACCACACTGC-3′	Ou et al., 2009
	R 5′-TGCACCCAGTTTTCCTTGG-3′	
GAPDH	F 5′-AGGTCGGAGTCAACGGATTT-3′	Schüller et al., 2009
	R 5′-TGGAAGATGGTGATGGGATTT-3′	
LL-37	F 5′-GTGCCCCAGGACGACACAGC-3′	This study
	R 5′-CCCCTGGCCTGGTTGAGGGT-3′	
Lysozyme	F 5′-AAAACCCCAGGAGCAGTTAAT-3′	Fahlgren et al., 2003
	R 5′-CAACCCTCTTTGCACAAGCT-3′	
POLR2A	F 5′-GATGGGCAAAAGAGTGGACTT-3′	Schüller et al., 2009
	R 5′-GGGTACTGACTGTTCCCCCT-3′	

*Forward (F) and reverse (R) primer sequences*.

### Scanning electron microscopy

Samples were fixed with 2.5% glutaraldehyde in PBS and dehydrated through graded acetone series. Specimens were dried using tetramethylsilane (Sigma), mounted on aluminum stubs, sputter-coated with gold (Polaron SC7640 sputter coater, Quorum Technologies), and viewed with a JEOL JSM 4900 LV scanning electron microscope.

### Immunofluorescence staining

Biopsy samples were fixed in 3.7% formaldehyde in PBS for 30 min, cryoprotected in 15 and 30% sucrose in PBS for 10 min, embedded in OCT compound (Sakura), snap-frozen in a dry ice/ethanol bath and stored at −70°C until use. Serial sections of 7 μm were cut with a Microm HM550 cryostat (Thermo Scientific), picked up on poly L-lysine-coated slides and air-dried. Tissue sections were blocked with 0.5% BSA in PBS for 20 min. Cryosections were subsequently incubated in rabbit anti-hBD2 (abcam) overnight at 4°C, washed and incubated in Alexa Fluor 488-conjugated secondary antibody (Life Technologies) for 30 min. Cell nuclei were counterstained with DAPI (Roche). Samples were mounted in Vectashield (Vector Laboratories) and analyzed using a fluorescence light microscope (Axiovert 200 M, Zeiss).

### IL-8 ELISA

Polarized T84 cells were lysed on ice in 1% Triton X-100 and 1μl/200 μl protease inhibitor cocktail (Sigma) in PBS, and Triton-insoluble proteins were removed by centrifugation. IL-8 concentrations in lysates and supernatants were determined using a human IL-8 ELISA kit (PeproTech) according to the manufacturer's instructions.

### Statistics

Statistical analysis was performed using GraphPad Prism software (version 5). qPCR data were log transformed before analysis. For parametric T84 cell data, one-way ANOVA with Tukey's multiple comparisons test was used to determine differences between multiple groups. For non-parametric biopsy data, Wilcoxon's signed-rank test or Kruskall–Wallis with Dunn's multiple comparisons test was used to determine differences between two or multiple groups, respectively. A *P* < 0.05 was considered significant.

## Results

### Apical EHEC infection of polarized T84 cells induces hBD2 and IL-8 expression

**Figure 1:**
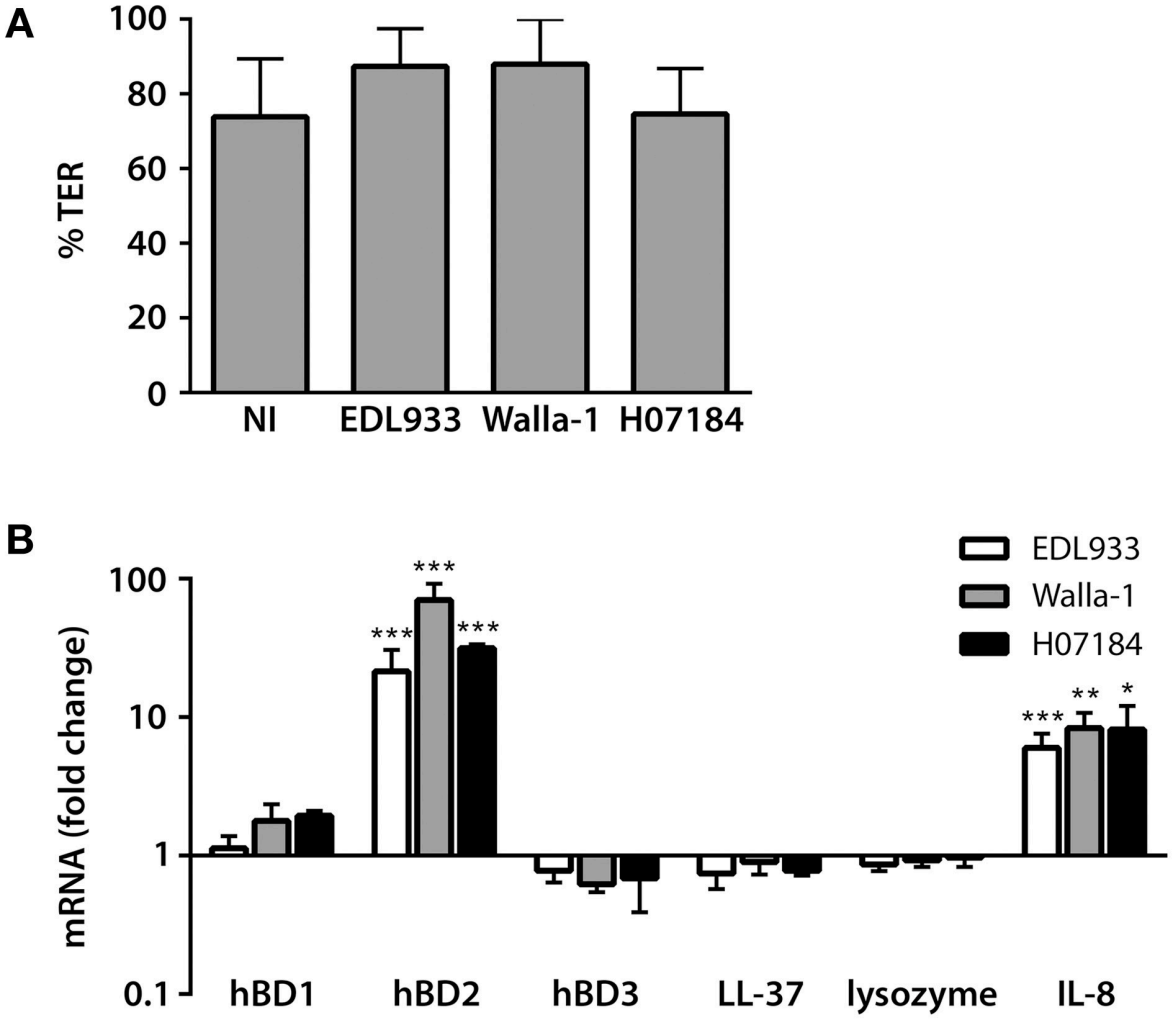
**EHEC infection induces hBD2 and IL-8 expression in polarized T84 cells**. Polarized T84 cells were apically infected with EHEC strains EDL933, Walla-1, H07184, or left non-infected (NI) for 9 h. **(A)** EHEC infection did not compromise epithelial barrier function. TER after infection is expressed as percentage of TER before infection. **(B)** AMP and IL-8 mRNA levels were quantified by qPCR and expressed as fold changes relative to NI controls. Data are shown as means ± standard errors of the means (SEM) of four independent experiments performed in duplicate. ^*^P < 0.05, ^**^P < 0.01, ^***^P < 0.001 vs. NI control.

To determine the influence of EHEC infection on AMP and IL-8 expression by human colonic epithelium, polarized T84 human colon carcinoma cells were infected with EHEC O157:H7 strains EDL933, Walla-1, and H0-7184-0336 (H07184) on the apical side. Bacterial overgrowth and loss of epithelial integrity was prevented by removal of bacteria after 3 h and subsequent medium exchange at hourly intervals. As shown in Figure 1A, this protocol resulted in maintenance of epithelial barrier function for up to 9 h as determined by TER. Expression of hBD1-4, LL-37, lysozyme, and IL-8 was determined by qPCR analysis. Results in Figure 1B show that apical EHEC infection resulted in a significant induction of hBD2 (21.5 ± 9.1 fold for EDL933, 70.4 ± 21.8 fold for Walla-1, and 31.5 ± 2.0 fold for H07184) and IL-8 (6.0 ± 1.6 fold for EDL933, 8.3 ± 2.4 fold for Walla-1, and 8.2 ± 3.9 fold for H07184) mRNA expression compared with non-infected (NI) controls whereas no significant change was observed for hBD1, hBD3, LL-37, and lysozyme. No specific amplification product was detected for hBD4. Subsequent kinetic analysis of hBD2 and IL-8 mRNA expression indicated that levels of both transcripts were first significantly induced at 6 h post-infection and showed a continuous increase during a 12 h period of infection (Supplementary Figure 1). For practical reasons, infections of polarized T84 cells were performed for 9 h in subsequent experiments.

### Apical EHEC infection results in hBD2 and IL-8 induction in a polarized human colonic biopsy model

**Figure 2:**
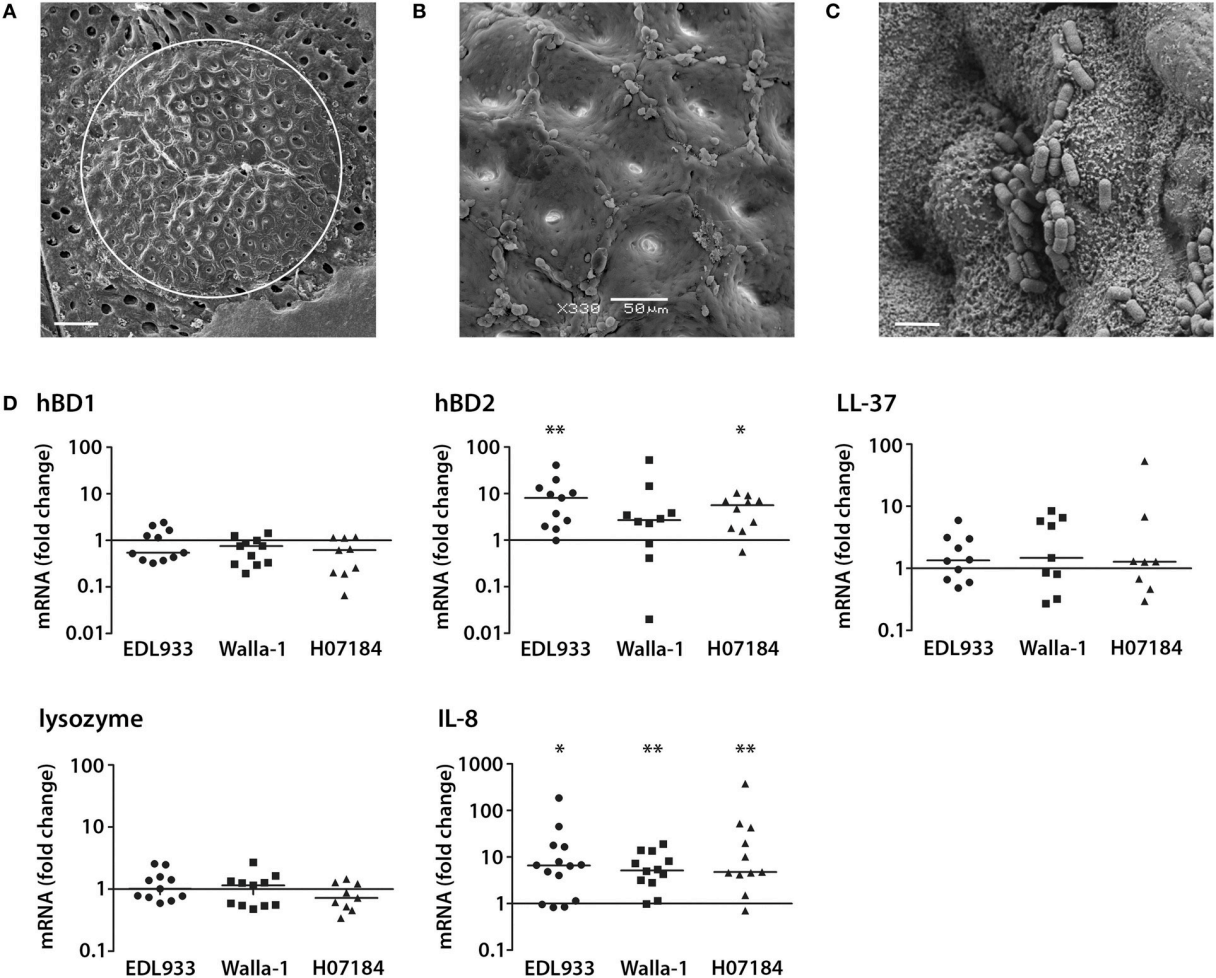
**EHEC induction of hBD2 and IL-8 in a polarized human colonic biopsy model**. Human colonic biopsy samples were sandwiched between two Perspex disks with a central aperture and mounted in Snapwell supports as described in Experimental Procedures. Mucosal sides of matched biopsies were infected with EHEC strains EDL933, Walla-1, H07184, or left NI for 7 h. **(A)** Scanning electron micrograph of colonic biopsy after 7 h of incubation. The white circle indicates the central aperture of the disk. Bar = 200 μm. **(B)** Intact colonic surface epithelium at higher magnification. Bar = 50 μm. **(C)** EDL933 intimately adhering to colonic biopsy epithelium. Bar = 2 μm. Images are representative of three experiments performed in duplicate. **(D)** AMP and IL-8 mRNA levels were quantified by qPCR and expressed as fold changes relative to matched NI controls. Data points represent individual biopsies from a total of six patients. Medians are indicated by a line. ^*^P < 0.05, ^**^P < 0.01 vs. NI control.

Our recent studies have demonstrated EHEC adherence to human colonic biopsy epithelium by forming typical A/E lesions (Lewis et al., 2015). To investigate the effect of EHEC infection on AMP and IL-8 expression *ex vivo*, a pIVOC model, which restricts bacterial access to the mucosal side of the biopsy, was developed. This was based on the pIVOC system established earlier to investigate the inflammatory response in duodenal biopsies (Schüller et al., 2009). Colonic biopsy samples were mounted in Snapwell supports, infected with EDL933, Walla-1, or H07184 on the mucosal side and incubated for up to 7 h. Tissue preservation and EHEC adherence were evaluated by scanning electron microscopy. As shown in Figure 2A, good tissue morphology was observed within the area of the central aperture (white circle) with intact surface epithelium visible at higher magnifications (Figure 2B). In addition, EHEC adhered intimately to colonic epithelium as described previously (Figure 2C; Lewis et al., 2015). Analysis of AMP and IL-8 expression by qPCR demonstrated a significant increase in hBD2 (median fold increase = 8.1 for EDL933 and 5.7 for H07184) and IL-8 expression (median fold increase = 6.6 for EDL933, 5.2 for Walla-1, and 4.8 for H07184) in EHEC-infected samples compared with non-infected controls. In contrast, transcript levels of hBD1, LL-37, and lysozyme were not significantly affected by EHEC infection (Figure 2D). Amplification levels for hBD3 and hBD4 were generally below detection threshold levels. However, amplification products of corresponding sizes were detected in some experiments, thereby confirming primer specificity and functionality of the qPCR assay (data not shown).

### EHEC induction of colonic epithelial hBD2 and IL-8 expression is dependent on flagellin

**Figure 3:**
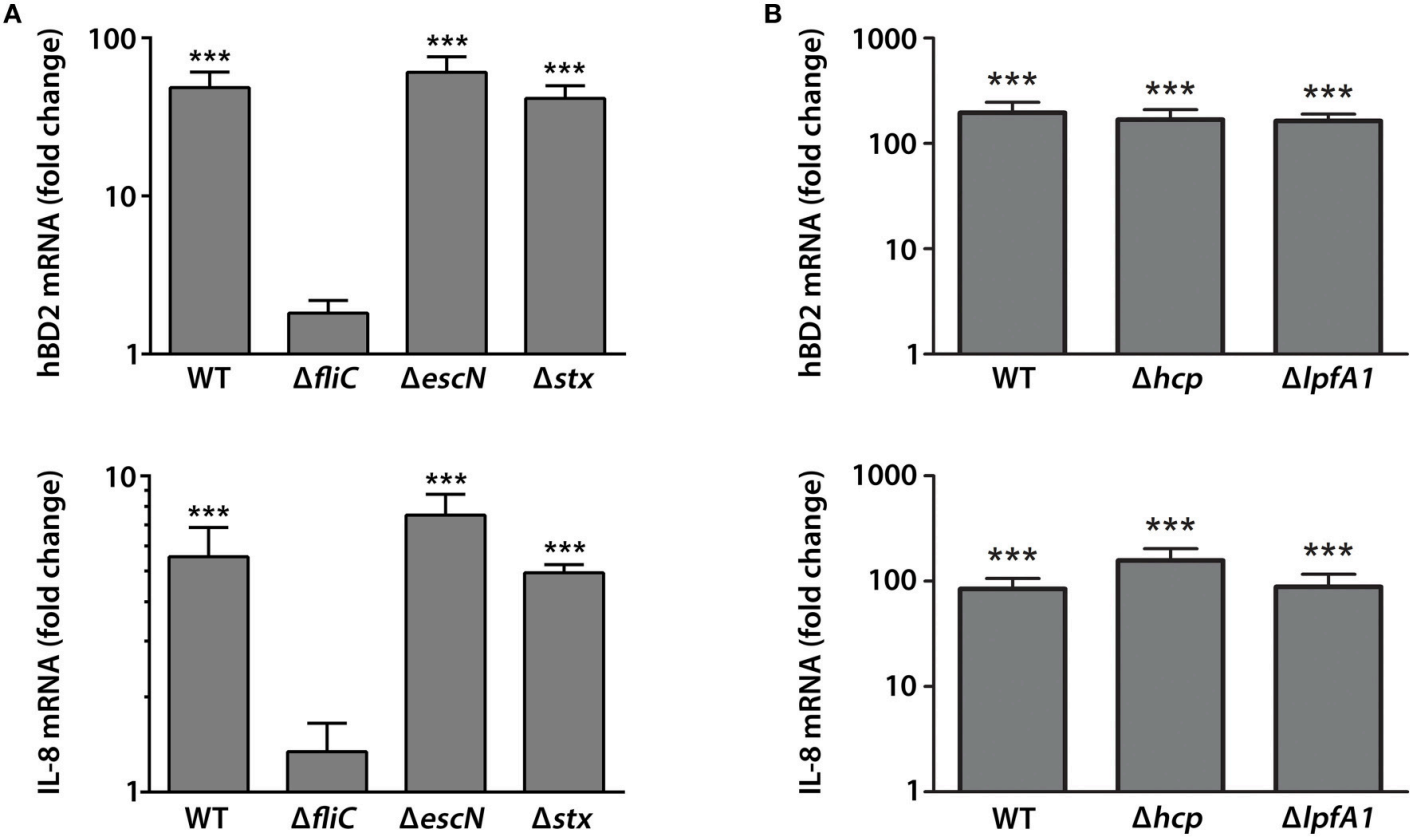
**EHEC induction of hBD2 and IL-8 expression in polarized T84 cells is dependent on flagellin**. Polarized T84 cells were apically infected with EHEC EDL933 wild-type (WT) and isogenic deletion mutants in *fliC, escN*, or *stx* **(A)** or EHEC 85–170 wild-type (WT) and isogenic deletion mutants in *hcp* or *lpfA1* **(B)** for 9 h. IL-8 and hBD2 mRNA levels were quantified by qPCR and expressed as fold changes relative to NI controls. Data are shown as means ± SEM of three independent experiments performed in duplicate. ^***^P < 0.001 vs. NI control.

We next determined which EHEC proteins were involved in hBD2 and IL-8 induction in colonic epithelium. Polarized T84 cells were apically infected with wild-type (wt) EDL933 or isogenic deletion mutants in *fliC* (flagellin, main component of EHEC flagellum), *escN* (cytoplasmic ATPase of T3SS), or *stx* (Stxs) for 9 h. In addition, the contribution of long polar fimbriae (Lpf) and hemorrhagic coli pili (HCP) was examined by using isogenic deletion mutants in *lpfA1* and *hcp* of EHEC strain 85–170. None of the strains significantly compromised epithelial barrier function as determined by TER (Supplementary Figure 2A). Expression levels of hBD2 and IL-8 were determined by qPCR. Whereas, infection with EDL933 wt, Δ*escN*, and Δ*stx* showed a significant induction of hBD2 (48.6 ± 12.3 fold for wt, 60.5 ± 15.6 fold for Δ*escN*, and 41.5 ± 8.3 fold for Δ*stx*) and IL-8 (5.6 ± 1.3 fold for wt, 7.5 ± 1.2 fold for Δ*escN*, and 4.9 ± 0.3 fold for Δ*stx*) expression vs. NI controls, no significant effect was observed in EDL933 Δ*fliC*-infected cells (Figure 3A). For infections with 85–170, all strains significantly increased expression of hBD2 (195.9 ± 50.1 fold for wt, 168.6 ± 40.4 fold for Δ*hcp*, and 163.4 ± 26.8 fold for Δ*lpfA1*) and IL-8 (84.3 ± 21.8 fold for wt, 157.5 ± 45.7 fold for Δ*hcp*, and 88.4 ± 28.0 fold for Δ*lpfA1*) vs. NI controls (Figure 3B). Notably, induction levels of hBD2 and IL-8 expression by EHEC strain 85–170 were considerably higher compared with EDL933 (Figure 3).

**Figure 4:**
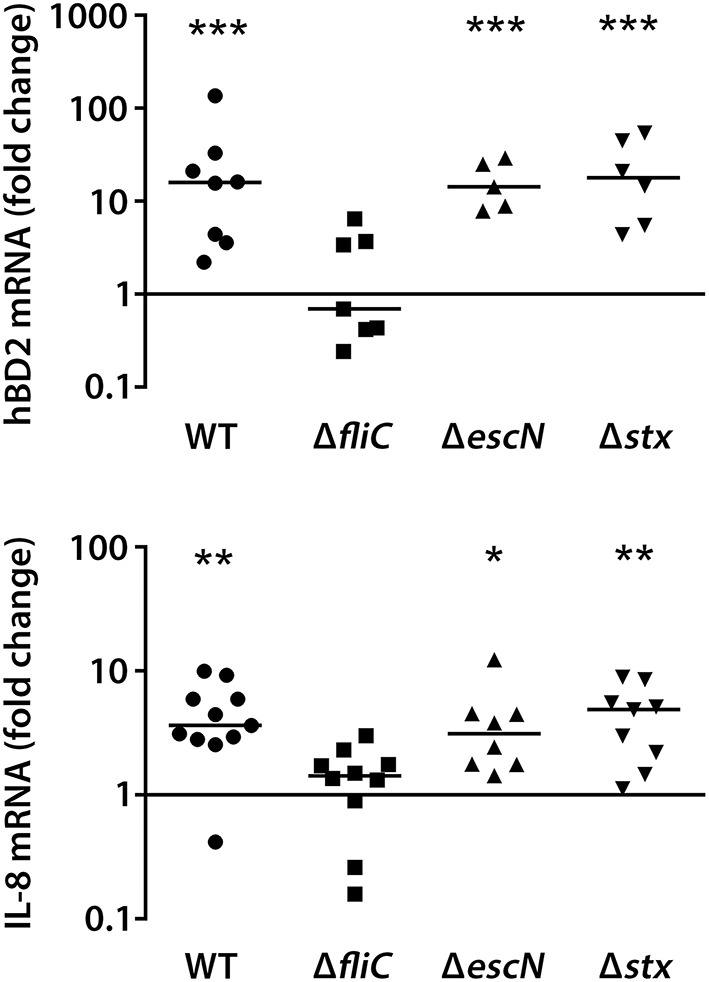
**EHEC stimulation of IL-8 and hBD2 expression in colonic biopsies requires flagellin**. Colonic biopsies were infected with EHEC EDL933 wild-type (WT), isogenic deletion mutants in *fliC, escN*, or *stx* or left NI on the mucosal side, and incubations were performed for 7 h. IL-8 and hBD2 mRNA levels were quantified by qPCR and expressed as fold changes relative to matched NI controls. Data points represent individual biopsies from a total of four patients. Medians are indicated by a line. ^*^P < 0.05, ^**^P < 0.01, ^***^P < 0.001 vs. NI control.

We also determined the effect of flagellin, T3S and Stxs on hBD2 and IL-8 expression in colonic pIVOC. As shown in Figure 4, EDL933 wt, Δ*escN*, and Δ*stx* significantly enhanced hBD2 (median fold increase = 15.9 for wt, 14.3 for Δ*escN*, and 17.8 for Δ*stx*) and IL-8 (median fold increase = 3.6 for wt, 3.1 for Δ*escN*, and 4.9 for Δ*stx*) expression in colonic biopsies whereas no significant effect was observed for the Δ*fliC* mutant.

### Purified flagellin induces hBD2 and IL-8 expression in colonic epithelium

**Figure 5:**
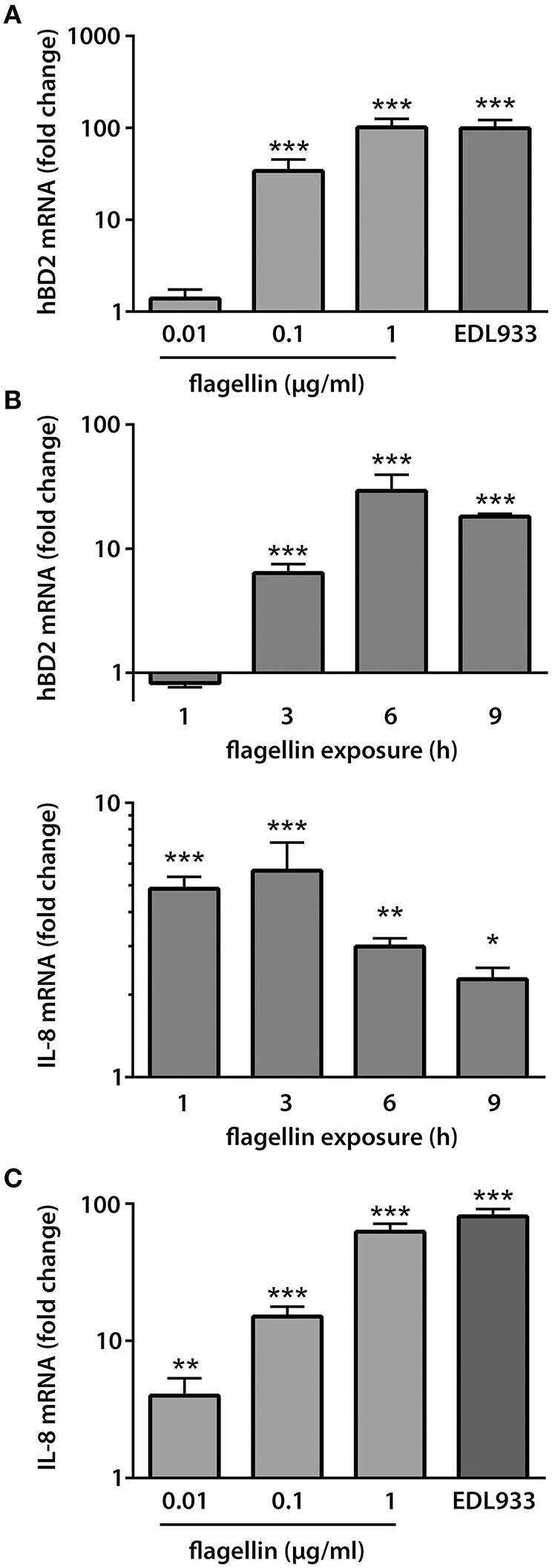
**EHEC flagellin is sufficient to induce hBD2 and IL-8 expression in polarized T84 cells**. Polarized T84 cells were incubated with EDL933, purified flagellin or left NI on the apical side. IL-8 and hBD2 mRNA levels were quantified by qPCR and expressed as fold changes relative to NI controls. **(A)** Purified flagellin induced a dose-dependent increase in hBD2 mRNA expression after 9 h. Data are shown as means ± SEM of three independent experiments performed in duplicate. **(B)** Kinetics of IL-8 and hBD2 induction by purified flagellin (0.1 μg/ml). Data are shown as means ± SEM of two independent experiments performed in duplicate. **(C)** Purified flagellin induced a dose-dependent increase in IL-8 mRNA expression after 2 h. Data are shown as means ± SEM of two independent experiments performed in duplicate. ^*^P < 0.05, ^**^P < 0.01, ^***^P < 0.001 vs. NI control.

To examine whether EHEC flagellin alone was sufficient to increase hBD2 and IL-8 expression, polarized T84 cells were infected with EDL933 or incubated with different concentrations of purified LPS-free monomeric H7 flagellin on the apical side for 9 h. No significant effect on TER was observed under any of the conditions tested (Supplementary Figure 2A). As demonstrated in Figure 5A, a dose-dependent increase in hBD2 mRNA expression was observed after 9 h of incubation with similar induction levels for 1 μg/ml flagellin and infection with EDL933 (101.5 ± 24.0 fold for flagellin and 99.0 ± 23.0 fold for EDL933). In contrast, only very low induction levels were noted for IL-8 (Figure 5B, data shown for 0.1 μg/ml flagellin). Therefore, kinetic analyses were performed, and cells were apically exposed to flagellin for 1–9 h. Whereas, highest induction levels for hBD2 were observed after 6–9 h of flagellin exposure, IL-8 mRNA expression was induced much earlier and peaked at 1–3 h of exposure (Figure 5B). IL-8 induction was subsequently examined after 2 h of flagellin exposure, and a dose-dependent response was noted with induction levels for 1 μg/ml flagellin approximating those observed during infection with EDL933 (62.6 ± 8.9 fold for flagellin and 80.8 ± 10.5 fold for EDL933; Figure 5C).

**Figure 6:**
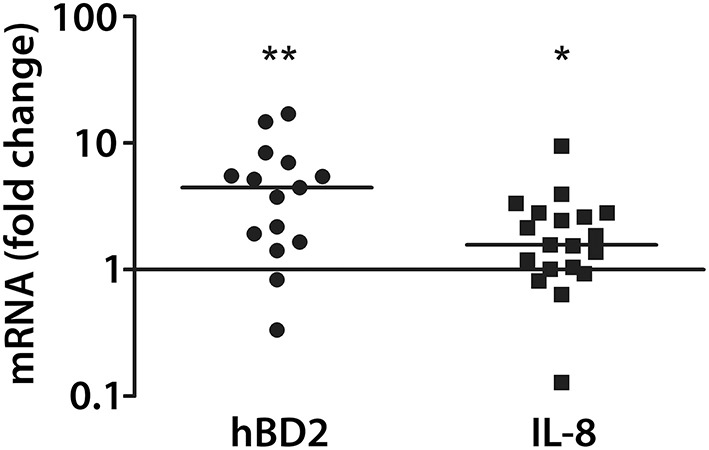
**EHEC flagellin induces hBD2 and IL-8 expression in colonic biopsies**. Colonic biopsies were incubated with purified flagellin (1 μg/ml) or left NI on the mucosal side for 7 h. IL-8 and hBD2 mRNA levels were quantified by qPCR and expressed as fold changes relative to matched NI controls. Data points represent individual biopsies from a total of four patients. Medians are indicated by a line. ^*^P < 0.05, ^**^P < 0.01 vs. NI control.

Similar to findings in polarized T84 cells, purified flagellin was also sufficient to induce hBD2 (median fold increase = 5.3) and IL-8 (median fold increase = 2.2) expression in colonic pIVOC (Figure 6).

### Flagellin-induced hBD2 expression is mediated by NF-κB, map kinases p38 and JNK

**Figure 7:**
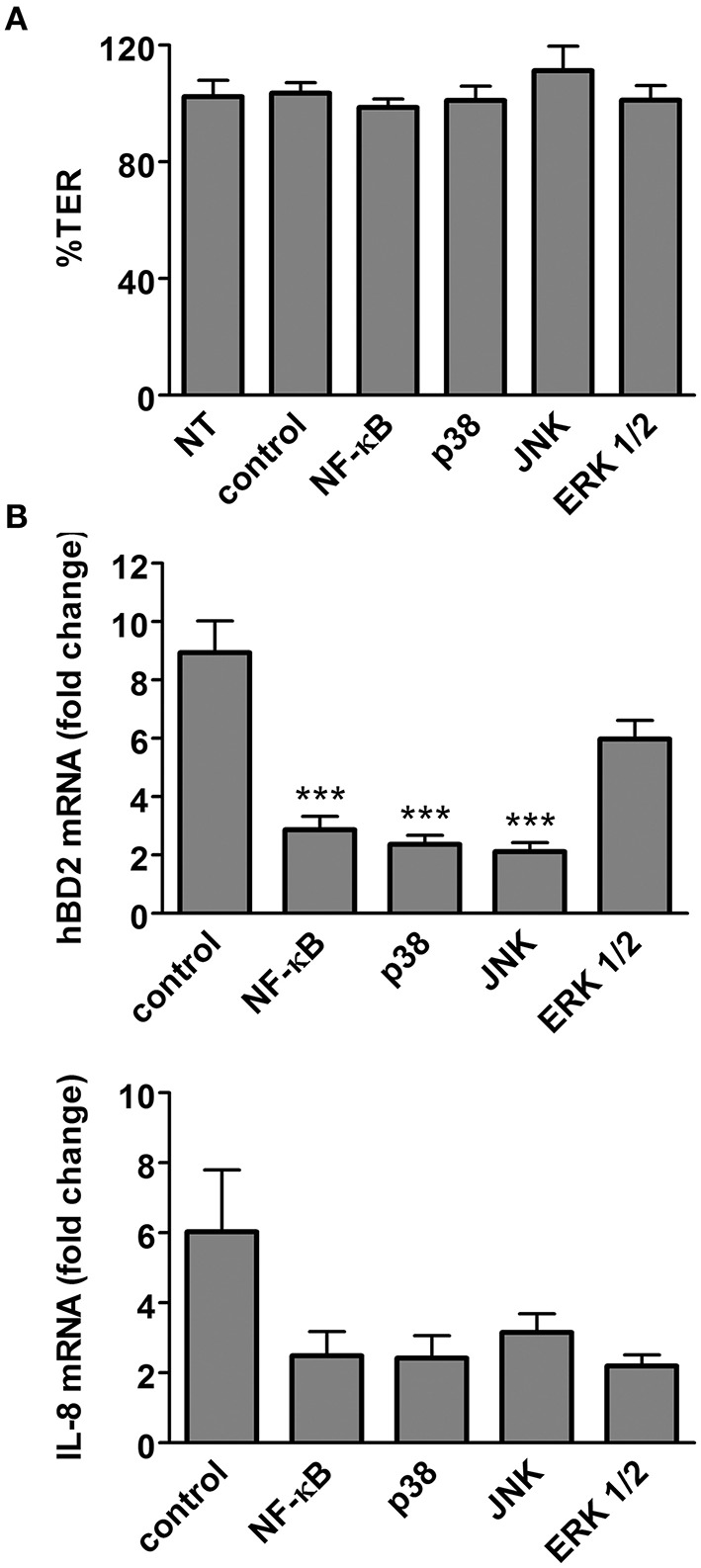
**Flagellin-mediated hBD2 induction is dependent on NF-κB, MAPK p38, and JNK**. Polarized T84 cells were incubated with specific inhibitors of NF-κB, p38, JNK, ERK1/2, or solvent (control) for 1 h before purified flagellin (0.1 μg/ml) was added for 6 h. Alternatively, T84 cells were left untreated (NT) for 7 h. **(A)** Influence of chemical inhibitors on epithelial barrier function. TER after treatment is expressed as percentage of TER before treatment. **(B)** hBD2 and IL-8 mRNA levels were quantified by qPCR and expressed as fold changes relative to NT samples. ^***^P < 0.001 vs. control. Data are shown as means ± SEM of three independent experiments performed in duplicate.

We further characterized the signaling pathways involved in flagellin-induced hBD2 and IL-8 expression. To this aim, polarized T84 cells were treated with specific chemical inhibitors before flagellin (0.1 μg/ml) was added. Gene expression was quantified by qPCR. None of the chemical inhibitors compromised epithelial barrier function (Figure 7A). While inhibition of NF-κB or the MAPKs p38 and JNK significantly reduced hBD2 gene expression, treatment with the ERK1/2 inhibitor did not have any significant effect (Figure 7B). For IL-8, all inhibitors reduced gene expression, but this did not reach significance (Figure 7B).

### EHEC infection induces epithelial hBD2 peptide expression in colonic biopsies

**Figure 8:**
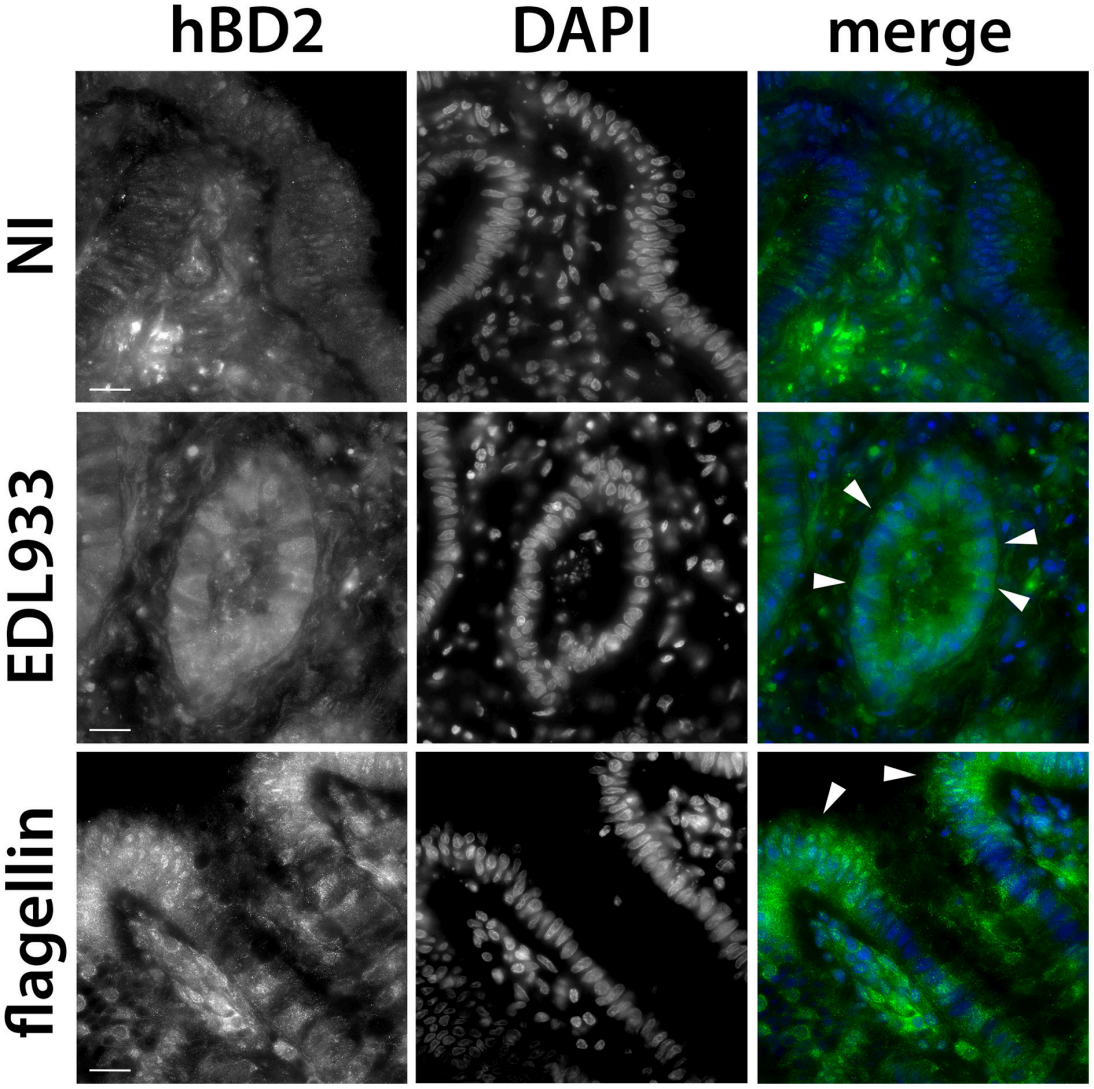
**EHEC stimulates hBD2 peptide expression in colonic biopsy epithelium**. Colonic biopsies were incubated with EDL933, purified flagellin (1 μg/ml) or left NI for 8 h. Cryosections from snap-frozen tissue were stained with anti-hBD2 (green), and cell nuclei were counterstained with DAPI (blue). Arrowheads show epithelial cells with induced hBD2 expression. Images are displayed as separate monochrome images of each fluorescence channel (hBD2, DAPI) and merged color images from both channels (merge). Shown are representative images from two independent experiments performed in duplicate. Bars = 10 μm.

Having shown that EHEC flagellin induced hBD2 mRNA expression, we investigated whether this also affected hBD2 peptide levels. Colonic biopsies were infected with EDL933 or incubated with purified flagellin (1 μg/ml) on the mucosal side, and hBD-2 peptide expression was assessed by immunofluorescence staining. As shown in Figure 8, elevated hBD2 peptide expression was observed in epithelial cells of colonic biopsies incubated with EDL933 or flagellin vs. NI controls.

### EHEC-induced IL-8 protein secretion in polarized T84 cells is directional and depends on the side of exposure

**Figure 9:**
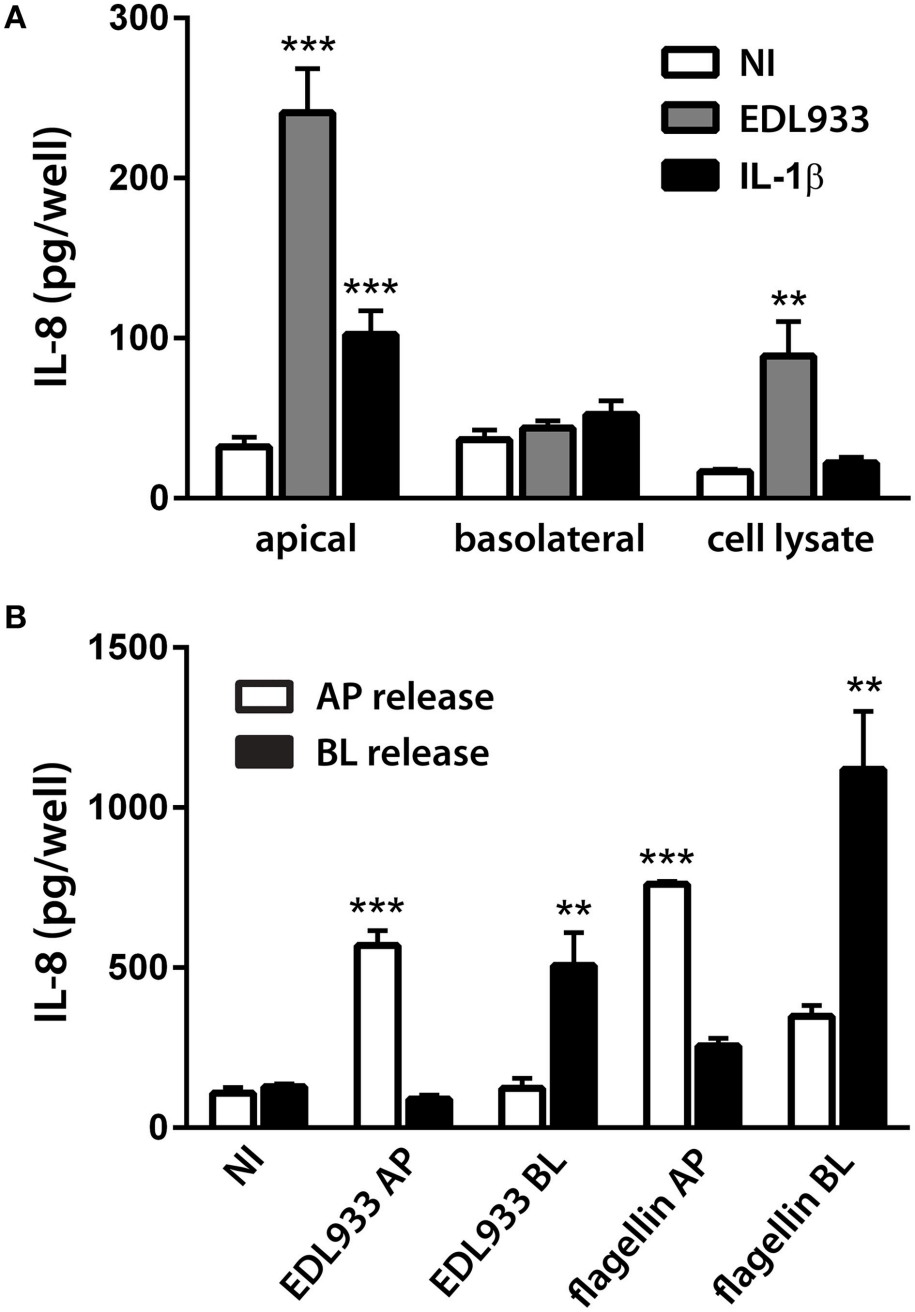
**EHEC-induced IL-8 release from polarized T84 cells is directional and depends on the side of exposure**. Polarized T84 cells were incubated with EDL933, IL-1β (10 ng/ml), flagellin (1 μg/ml), or left NI on the apical **(A,B)** or basolateral side **(B)** for 24 h. IL-8 protein levels in cell lysates, apical and basolateral supernatants were determined by ELISA. **(A)** Apical exposure to EDL933 or IL-1β stimulated apical IL-8 release. Data are shown as means ± SEM of three independent experiments performed in duplicate. **(B)** Apical (AP) exposure to EDL933 or flagellin induced apical IL-8 release (white columns), whereas basolateral (BL) exposure to EDL933 or flagellin induced basolateral IL-8 release (black columns). Data are shown as means ± SEM of two independent experiments performed in triplicate. ^**^P < 0.01, ^***^P < 0.001 vs. NI control.

We further investigated EHEC-induced IL-8 expression at the protein level. Polarized T84 cells were apically infected with EDL933 or incubated with the potent IL-8 inducer IL-1β (10 ng/ml) for 24 h. This time point was chosen according to previous IL-8 secretion studies on EHEC and enteropathogenic *E. coli* (EPEC) (Hauf and Chakraborty, 2003; Baruch et al., 2011; Sham et al., 2011). To prevent cell damage and breach of barrier function due to bacterial overgrowth and acidification, EHEC-infected monolayers were treated with gentamicin (200 μg/ml) after 1 h. This treatment did not result in complete killing, but reduced bacterial growth so that infections could be continued for up to 24 h. TER was not significantly affected by any of the treatments (Supplementary Figure 2B) indicating maintenance of epithelial barrier function. At the end of the incubation, IL-8 protein levels were determined in cell lysates and apical and basolateral supernatants. As shown in Figure 9A, apical EHEC infection significantly increased IL-8 protein levels in apical supernatants (240.9 ± 27.5 vs. 32.0 ± 6.2 pg/well in NI controls) and to a lesser extent in cell lysates (88.9 ± 21.5 vs. 16.6 ± 1.4 pg/well in NI controls) but not in basolateral media. Similarly, apical IL-1β exposure resulted in significantly enhanced amounts of IL-8 in apical supernatants (102.3 ± 14.9 vs. 32.0 ± 6.2 pg/well in NI controls) but not in cell lysates or basolateral media. We further extended these studies and investigated the influence of the side of exposure on the direction of IL-8 release into the media. To this aim, polarized T84 cells were incubated with EDL933 or purified flagellin (1 μg/ml) on the apical or basolateral side for 24 h, and IL-8 levels were evaluated in apical and basolateral supernatants. None of the treatments significantly affected the TER (Supplementary Figure 2B). As demonstrated in Figure 9B, apical exposure to EDL933 or flagellin led to a significant increase of IL-8 secretion into apical supernatants (569.5 ± 46.2 or 761.2 ± 8.5 vs. 108.2 ± 16.9 pg/well in NI controls, respectively) whereas IL-8 levels in basolateral media were not significantly affected. On the other hand, basolateral exposure of polarized T84 cells to EDL933 or flagellin significantly stimulated IL-8 protein release into basolateral compartments (506.6 ± 103.2 or 1119.0 ± 181.4 vs. 128.6 ± 8.1 pg/well in NI controls, respectively) whereas IL-8 levels in apical media were not significantly affected (Figure 9B).

## Discussion

The intestinal epithelium is the first line of defense against enteric pathogens, and the expression and release of AMPs represents an important part of the innate immune response during intestinal infections. In the colon, the main target site of EHEC, epithelial cells express hBD1-4, LL-37, and lysozyme. In this study, we have investigated the effect of EHEC infection on AMP expression in two physiologically relevant infection models: human colonic biopsies and polarized T84 cells. As demonstrated by our recent studies, IVOC of human colonic biopsies supports EHEC A/E lesion formation (Lewis et al., 2015). However, standard IVOC is not suitable to study the host immune response as it allows bacterial access and stimulation of the submucosal tissue surface. Therefore, we adopted a polarized IVOC method developed previously for small intestinal biopsy samples (Schüller et al., 2009). In addition to IVOC, we used T84 human colon carcinoma cells which lack expression of the Stx receptor Gb3 similar to human colonic epithelium (Schüller et al., 2004) and form highly polarized epithelia with high TER when grown on membrane supports (Madara et al., 1987).

Our results show that apical EHEC infection induced hBD2 expression in both colonic biopsies and polarized T84 cells whereas all other AMPs remained unaffected. This is of relevance as hBD2 has been demonstrated to directly kill *E. coli* and also promote the release of immuno-modulating adenosine (Estrela et al., 2013). In contrast to hBD1 which is constitutively expressed in human colonic epithelium and thus confers a baseline protection against bacterial pathogens, hBD2 is undetectable or only expressed at very low levels in healthy children and adults, but elevated in patients with inflammatory bowel disease (Fahlgren et al., 2003; Zilbauer et al., 2010). This might be due to enhanced contact of the gut microbiota with intestinal epithelium, and previous studies have demonstrated induction of hBD2 by both commensal and pathogenic bacteria. Most of these studies were performed using intestinal epithelial cell lines (e.g., Caco-2 and HT-29) and have demonstrated stimulation of hBD2 expression by enteroinvasive pathogens such as *Salmonella* spp., *Shigella flexneri* and *dysenteriae*, enteroinvasive *E. coli*, and *Campylobacter jejuni* (O'Neil et al., 1999; Ogushi et al., 2001; Hase et al., 2002; Zilbauer et al., 2005). In addition, non-invasive EPEC and probiotic *E. coli* Nissle 1917 have also been shown to induce hBD2 expression (Schlee et al., 2007; Khan et al., 2008). In contrast, very few studies have been carried out on human intestinal tissue so far. Whereas experiments using human intestinal xenografts in mice have confirmed hBD2 induction by *Salmonella typhi* (O'Neil et al., 1999), no change in expression was observed after infection with *S. flexneri* (Sperandio et al., 2008). To the best of our knowledge, this is the first study using IVOC of human intestinal biopsies to investigate the AMP response to bacterial infection.

We further investigated the bacterial factors involved in hBD2 induction and found that EHEC flagellin was both necessary and sufficient to stimulate a response *ex vivo* and *in vitro*. This is in agreement with studies on EPEC and *Salmonella enterica* serotype Enteritidis which also demonstrated hBD2 induction mediated by flagellin (Ogushi et al., 2001; Schlee et al., 2007; Khan et al., 2008). Interestingly, the hBD2 response to flagellin from non-pathogenic *E. coli* such as ATCC 259922, JM109, and Nissle 1917 is strain-dependent suggesting differences in binding to a potential receptor (Schlee et al., 2007). We further tested which signal transduction pathways were involved in flagellin-mediated hBD2 induction by using chemical inhibitors of NF-κB and MAPKs. These studies showed that flagellin-induced hBD2 expression was dependent on NF-κB, MAPK p38, and JNK but not ERK1/2. NF-κB dependency of hBD2 induction has been described before in LPS-stimulated macrophages (Tsutsumi-Ishii and Nagaoka, 2002) and intestinal epithelial cells treated with flagellin from *E. coli* Nissle (Wehkamp et al., 2004). The latter study also demonstrated involvement of the MAPKs JNK but not p38 or ERK 1/2. In addition, *Bacteroides fragilis* enterotoxin induced hBD2 by p38 activation (Yoon et al., 2010), and inhibitors of p38, JNK and ERK 1/2 reduced hBD2 induction by probiotic lactobacilli, although this did not reach significance (Schlee et al., 2008).

In addition to studying the AMP response to EHEC infection, we also examined expression of the pro-inflammatory cytokine IL-8 which acts as a chemoattractant and recruits neutrophils to the site of infection (Baggiolini and Clark-Lewis, 1992). Previous studies using non-polarized cells have shown that EHEC infection induces IL-8 release, and that this is dependent on NF-κB and MAP kinases (Dahan et al., 2002; Gobert et al., 2007). While several bacterial factors including long polar fimbriae, hemorrhagic coli pili, and Shiga toxins (Thorpe et al., 2001; Ledesma et al., 2010; Farfan et al., 2013) have been implicated in this response, many studies suggest that flagellin is the principal inducer of IL-8 production during EHEC infection (Berin et al., 2002; Zhou et al., 2003; Miyamoto et al., 2006). This is confirmed by our results on human colonic biopsies and polarized T84 cells and agrees with earlier *ex vivo* studies using human colonic xenografts in mice (Miyamoto et al., 2006).

While flagellin provides the initial stimulus for a pro-inflammatory response, it has been shown that T3S effectors encoded outside the locus of enterocyte effacement dampen down NF-κB activation and IL-8 release at later stages of EHEC and EPEC infection (Hauf and Chakraborty, 2003; Nadler et al., 2010; Baruch et al., 2011). In addition, a similar T3S-dependent suppressive effect has been observed in EPEC-dependent hBD2 expression (Khan et al., 2008). Notably, all of these studies have been performed using cervical HeLa cells or undifferentiated Caco-2 cells. Interestingly, we did not detect any significant inhibitory effect of the T3SS on hBD2 and IL-8 expression during EHEC infection of colonic biopsies and polarized T84 cells. A similar observation has been reported by Ruchaud-Sparagano and colleagues who demonstrated T3S-dependent suppression of IL-8 induction in EPEC-infected polarized Caco-2 but not T84 cells (Ruchaud-Sparagano et al., 2007). As fully differentiated Caco-2 cells display a small intestinal rather than colonic phenotype (Engle et al., 1998), these results might suggest a delayed or absent immunosuppressive effect of EHEC infection in human colonic vs. small intestinal epithelium.

As it was technically not feasible to accurately quantify epithelial IL-8 secretion in colonic biopsies due to the presence and varying thickness of the submucosal tissue, polarized T84 cells were employed for this part of the study. Unexpectedly, we found that IL-8 secretion was vectorial and depended on the direction of the stimulus: Whereas, apical exposure to EHEC or flagellin induced apical IL-8 release, basolateral exposure resulted in IL-8 secretion to the basolateral side. Whilst EHEC-induced IL-8 secretion has not been examined in polarized T84 cells, studies on EPEC and EPEC flagellin have reported increased basolateral IL-8 secretion after basolateral but not apical stimulation (Zhou et al., 2003; Ruchaud-Sparagano et al., 2007). Very few studies have examined apical IL-8 secretion as it is generally assumed that IL-8 is mainly secreted basolaterally to mediate neutrophil recruitment to the epithelium. In line with this theory, predominantly basolateral IL-8 secretion has been shown in polarized HCA-7 cells stimulated with EHEC flagellin and polarized Caco-2 cells apically exposed to EPEC (Berin et al., 2002; Ruchaud-Sparagano et al., 2007). However, similar to our results, stimulation of polarized HT 29/19A or Caco-2 cells with IL-1 or TNF-α resulted in polarized IL-8 secretion dependent on the side of exposure (Lammers et al., 1994; Sonnier et al., 2010). In addition, vectorial IL-8 release has been shown in flagellin-stimulated polarized Caco-2 cells (Rossi et al., 2013). While luminal IL-8 could aid neutrophil transmigration across the epithelium as demonstrated in urinary tract infections (Godaly et al., 2001), autocrine epithelial signaling via apically expressed IL-8 receptors (CXCR1) has also been suggested (Rossi et al., 2013).

Taken together, our study demonstrates that colonic EHEC infection elicits a weak innate immune response with induction of hBD2 but no other AMPs and low levels of IL-8. These results suggest that inflammation plays a minor role in intestinal EHEC pathogenesis.

## Author contributions

SL and SS designed the study, analyzed the data, and prepared the manuscript. SL and SE performed the experimental work. AP, VC, SC, and WG selected suitable patients, obtained informed consent, and provided human biopsy samples.

### Conflict of interest statement

The authors declare that the research was conducted in the absence of any commercial or financial relationships that could be construed as a potential conflict of interest.

## Supplementary material

Supplementary Figure 1 **Kinetics of hBD2 and IL-8 gene expression during EHEC infection**. Polarized T84 cells were apically infected with EHEC strain EDL933 for 3–12 h, or left NI for 12 h. hBD2 and IL-8 mRNA levels were quantified by qPCR and expressed as fold changes relative to NI controls. Data represent means ± SEM of two independent experiments. ^*^*P* < 0.05, ^**^*P* < 0.01, ^***^*P* < 0.001 vs. NI control.

Supplementary Figure 2 **Influence of different treatments on T84 cell barrier function**. Polarized T84 cells were apically inoculated with EHEC wild-type EDL933 or 85–170 (WT), deletion mutants (Δ*fliC, escN, stx, hcp*, or *lpfA1*), purified flagellin (1 μg/ml) or left non-infected (NI) for 9 h **(A)** or incubated with EDL933, IL-1β (10 ng/ml), flagellin (1 μg/ml), or left NI on the apical (AP) or basolateral side (BL) for 24 h **(B)**. TER after treatment is expressed as percentage of TER before treatment. Data are shown as means ± SEM of three independent experiments performed in duplicate.
