# Supplementary figures and images for: Flagellin Induces β-Defensin 2 in Human Colonic Ex vivo Infection with Enterohemorrhagic Escherichia coli
Source: Front Cell Infect Microbiol. 2016 Jun 21;6:68. doi: 10.3389/fcimb.2016.00068 (PMC4914554; doi:10.3389/fcimb.2016.00068)

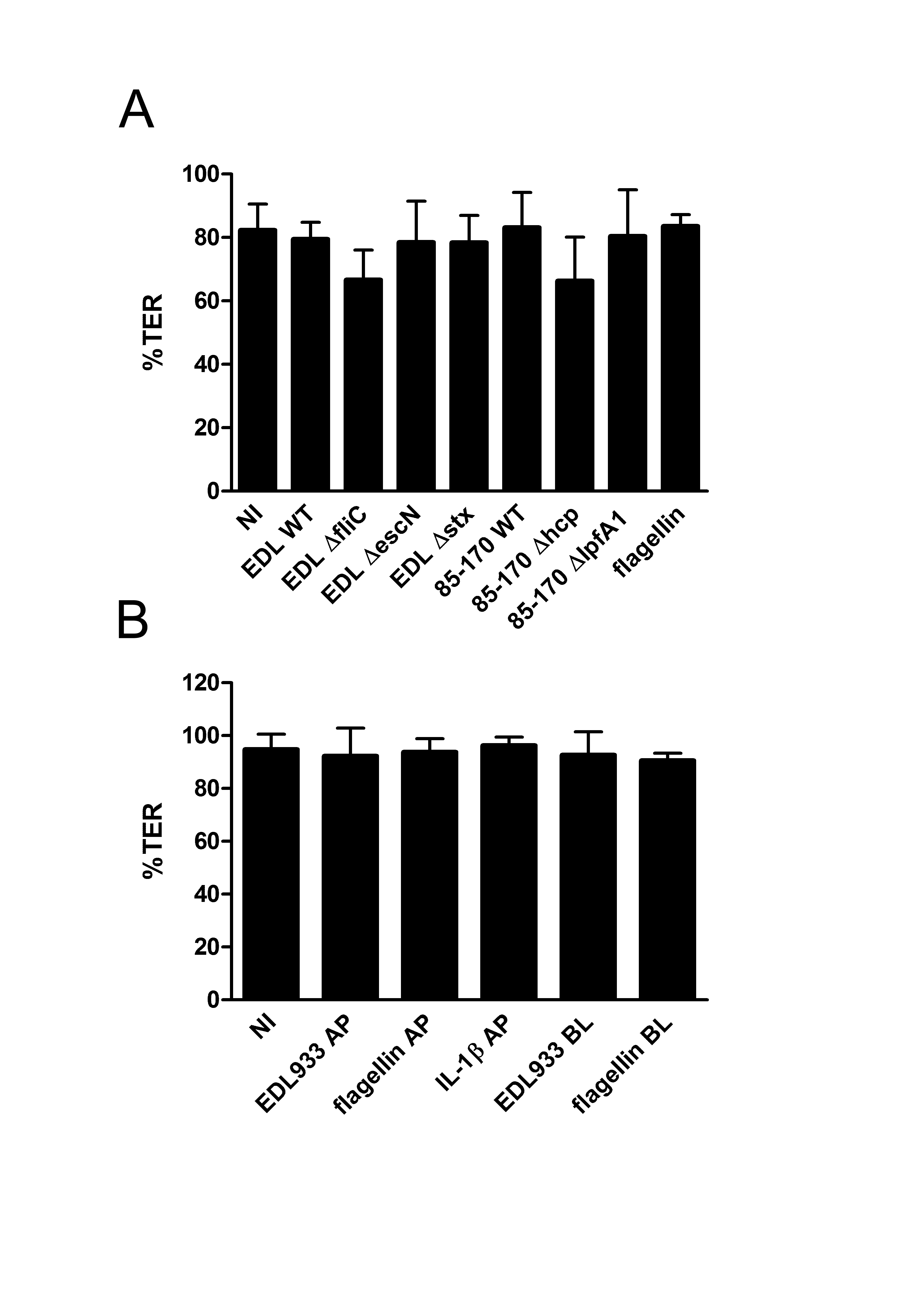

Supplement: Supplementary Figure 2 — Influence of different treatments on T84 cell barrier function. Polarized T84 cells were apically inoculated with EHEC wild-type EDL933 or 85–170 (WT), deletion mutants (ΔfliC, escN, stx, hcp, or lpfA1), purified flagellin (1 μg/ml) or left non-infected (NI) for 9 h (A) or incubated with EDL933, IL-1β (10 ng/ml), flagellin (1 μg/ml), or left NI on the apical (AP) or basolateral side (BL) for 24 h (B). TER after treatment is expressed as percentage of TER before treatment. Data are shown as means ± SEM of three independent experiments performed in duplicate. [file Image2.TIF]
